# The Mechanisms Underlying *Helicobacter Pylori*-Mediated Protection against Allergic Asthma

**Published:** 2017-06

**Authors:** Kamran Bagheri Lankarani, Behnam Honarvar, Seyyed Shamsadin Athari

**Affiliations:** 1 Health Policy Research Center, Institute of Health, Shiraz University of Medical Sciences, Shiraz, Iran; 2 Department of Immunology, Faculty of Medicine, Zanjan University of Medical Sciences, Zanjan, Iran; 3 Research Center for Food Hygiene and Safety, Shahid Sadoughi University of Medical Sciences, Yazd, Iran

**Keywords:** Helicobacter pylori, Allergic Asthma, Protection

## Abstract

*Helicobacter pylori*, a gram negative pathogen, infects the stomach and gastrointestinal tract and causes pathological damage to these organs. *H. pylori* infection is more prevalent among people living in developing countries. Allergic asthma is a chronic inflammatory disease of the airways. Hyperinflation, hyperresponsiveness, and abnormal immunological and inflammatory processes in respiratory airways typically occur during an asthma attack. The results of recent studies have suggested an association between *H. pylori* and asthma risk. However, the role of *H. pylori* infection in the pathophysiology of asthma is still a matter of debate. The results of some studies indicate an association between *H. pylori* infection and protection against allergic asthma. Exposure to infectious agents might educate the immune system and provide protection against allergic diseases. *H. pylori* inflammation also changes gastric hormonal levels and could influence the autonomic nervous system. T-regs could be influenced by the immunological response to *H. pylori* and then inhibit the Th-2-mediated allergic response. Therefore, *H. pylori* might play a protective role against asthma. *H. pylori* can also reduce gastro-esophageal reflux, which is an asthma stimulator. High loads of *H. pylori* are not always present during infection. It is not definitely clear whether *H. pylori* is a pathogen or simply an opportunist. It has been suggested that early exposure to *H. pylori* prevents development of pediatric asthma. Therefore, it is possible that therapeutic products made from *H. pylori* can be used for the treatment or prevention of asthma.

## INTRODUCTION

*Helicobacter pylori* (*H. pylori*) is a helically shaped, micro-aerophilic, and gram-negative bacterium that chronically infects the stomach of 45% of the human population. It causes pathologic effects in the stomach and the duodenum. Its presence increases the risk for peptic ulceration, gastric lymphoma, and adenocarcinoma (gastric cancer) (1–4). *H. pylori* may also contribute to idiopathic thrombocytopenic purpura and iron and vitamin B12 deficiencies in children. Based on epidemiologic information, people living in developing countries are more likely to acquire *H. pylori* infection (5–7).

However, the influence of *H. pylori* infection on diseases in other organs such as the respiratory system are not fully understood to date. There is some evidence that infection with *H. pylori* might be associated with the development of bronchiectasis, bronchitis, and lung cancer (7–9), but it seems that *H. pylori* infection has no direct role in the development and pathophysiology of bronchial asthma (8–10). In fact, an inverse relationship between *H. pylori* infection and the occurrence of allergic asthma has been reported (10–12).

Allergic asthma is a chronic inflammatory disease of the airways that is characterized by wheezing, coughing, and shortness of breath. Asthma is caused by an exaggerated Th2-mediated immune response to environmental allergens. Allergic asthma is characterized by a Th2 pattern of cytokines and inhibited secretion of IL-12 and IFN-γ (Th1 pattern of cytokines). Type 2 cytokines promote immunoglobulin (Ig) E production and eosinophilic inflammation in the airways (13–15).

Microorganisms (such as bacteria and viruses) that are responsible for infection in the respiratory system might play complex roles in the development of asthma as stimulatory or inhibitory agents (16, 17). Respiratory viral infections (such as influenza virus) could trigger the pathogenesis of aeroallergens. Conversely, some microbial-derived particles show adjuvant and anti-atopic activity (6, 17, 18).

In susceptible patients, triggering a Th1-mediated immune response could suppress Th2 responses while lack of an adequate Th1 response might result in an overactive Th2 response and allergic asthma in the airways (15). Microbial products have demonstrated a strong stimulatory effect that is helpful to maintain the balance between Th1 and Th2 responses. The microbes that are effective in controlling and treating asthma could be employed as potential therapeutic tools (6, 15, 18).

Recent studies have shown a correlation between the presence of *H. pylori* and a decreased risk of asthma. However, the results reported by different studies are contradictory (19–21). The prevalence of allergic asthma has increased considerably in developed countries. In developed countries, a correlation between allergic asthma and childhood infection with *H. pylori* is notable (20, 21). The present review will discuss *H. pylori* infection and its possible connection with allergic asthma. Moreover, potential therapeutic strategies to use *H. pylori* as a new tool for the prevention and treatment of allergic asthma will also be reviewed.

### Microbes and immune system responses

The immune system reacts against different microbes and produces specific responses for the specialized recognition and elimination of different infectious agents (16, 22).

Endogenous antigens such as viruses can be eliminated by CD8+ T cells (cytotoxic T lymphocytes) with MHC class I molecules via a cell-mediated cytotoxicity mechanism. Exogenous microbial antigens such as most bacteria activate the MHC class II possessing CD4+ T cells (helper T lymphocytes). The CD4+ T cells then stimulate B lymphocytes to produce antibodies (humoral-mediated immunity) for neutralization or elimination of extracellular microbes (16, 23, 24).

Different genetic and environmental factors in the microenvironment of the responding naïve T helper lymphocytes influence the differentiation of Th subsets by determining the cytokine orchestration of each Th subset. Th cell subsets are characterized by their ability to produce different cytokines and are polarized to the main subsets of Th1 and Th2 cells. If the exogenous antigen is immunogenic then subset 1 of T helper cells will be activated and release type 1 cytokines (IL-12, TNF-α, and INF-γ) and stimulate B cells to produce IgG for the elimination of antigens. If the exogenous antigen is an allergen, subset 2 of T helper cells will release type 2 cytokines (IL-4, IL-5, and IL-13) and stimulate B cells to produce IgE, which activates early type hyper-responsivity and leads to allergic reactions and atopy symptoms (16, 24–26).

### Allergic asthma

Asthma as a complicated chronic inflammatory airways disease is characterized by mucus hyper-secretion in the epithelium of the airway, showing typical airway hyperresponsiveness in response to allergen stimuli. Allergic asthma is a common multifactorial disease in childhood but can also develop at any age. Allergic asthma is an important public health problem that reduces the quality of life. Asthma is also associated with high direct and indirect healthcare costs. Asthma in older patients may be exacerbated by viral and bacterial pathogens instead of allergens (27–29). Prevention, recognition, control, and treatment of asthma are necessary for all populations.

An asthma attack is an inevitable result of improper functioning of the immune system. In histopathological evaluation, accumulation and infiltration of eosinophils indicating bronchial inflammation can be seen. In allergic asthma, IL-4 plays a critical role in antibody class switching to IgE (30–32). IL-5 is the most important cytokine in the eosinophilic inflammation process, while IL-13 stimulates mucus secretion (33, 34).

### Helicobacter pylori

*H. pylori* colonizes the human stomach in childhood and can persist for decades. Its helical shape and flagella allows it to corkscrew through the gastric mucus gel and adhere to the epithelium. *H. pylori* produces a cytoplasmic urease that neutralizes gastric acid in the periplasm (35–38).

*H. pylori* infection as an exogenous antigen induces a Th1 immune response in the mucosal layer of the gastrointestinal tract (GI) that leads to chronic gastric inflammation (39, 40). *H. pylori* neutrophil-activating protein (HP-NAP), a toll-like receptor (TLR) 2 activating protein, can strongly stimulate neutrophils, monocytes, and dendritic cells, and induce up-regulation of type 1 cytokines, thus promoting a polarized Th1 activation and response (41–43). Predominant activation of Th1 lymphocytes by *H. pylori* leads to the production of IFN-γ, IL-12, and TNF-α in the stomach (44–47).

HP-NAP has structural homology to the DNA protecting protein under starved conditions (Dps) family in that their structure consists of 12 identical subunits arranged in a dodecameric shell and 32 symmetric units (46, 48). In addition, in the inner part of the shell, 12 ferrous (iron) ions are bound and possibly represent sites for iron oxidation. This arrangement (quaternary) leads to resistance of the protein against denaturation (47, 49).

The extracellular domain of TLR2 is a cell surface innate immune receptor for HP-NAP that also functions in association with TLR4. TLR4 itself forms a complex with MD-2 to recognize pathogen antigens such as LPS (47, 50, 51). Binding of ligands to these extracellular domains induces the recruitment of specific adaptor proteins to their intracellular domains (52). Because ligands of TLR2 share structural similarity to the ligands of TLR4, HP-NAP may also activate the TLR4-MD2 complex. TLR2 and TLR4 induction is dependent on the activation of mitogen-activated protein kinases by phosphorylation of p38, JNK, and the activation of transcription factor NF-κB in immune cells. Therefore, TLR2 and TLR4 agonists can induce the Th1 type of immune response and cytokines production (45, 53).

### *Helicobacter pylori* and allergic asthma

According to epidemiological studies and the hygiene hypothesis, infectious diseases can influence the development of allergic disorders (54). Infections can inhibit allergic Th2 responses by directing the immune balance toward domination of the Th1 responses by production of IFN-γ and IL-12 (55, 56). Therefore, development of asthma can be prevented by administration of bacteria or their components, which induce Th1 responses (57).

Interestingly, an association between *H. pylori* infection and a decreased risk of allergic asthma has been proposed (8, 58). HP-NAP increases IFN-γ production and decreases IL-4, resulting in a redirection of the Th2 immune response toward a Th1 response. It has been proposed that HP-NAP might be responsible for the reduced allergic asthma frequency seen in *H. pylori*-infected patients (59). HP-NAP as an immunomodulator could be administered intraperitoneally or intranasally and could be used as a beneficial therapeutic agent for the prevention and treatment of allergic asthma.

Systemic administration of HP-NAP significantly reduces the serum levels of IgE, IL-5, and IL-4; increases IL-12 and IFN-γ levels (12, 60); and prevents eosinophil accumulation in airways (61). *H. pylori* up-regulates TLR4 and thus may play a protective role against allergic asthma. Furthermore, HP-NAP administration inhibits the Th2-mediated allergic inflammation of the bronchia in asthma. Thus, the increased prevalence of allergic asthma in western countries may be related to a decreased incidence of *H. pylori* infection. Therefore, it is possible that HP-NAP could be employed as an important candidate for prevention and treatment of allergic asthma (5, 58, 62, 63).

In summary, according to the hygiene hypothesis, exposure to infectious agents in an unhygienic environment might educate the immune system and protect against the development of allergic diseases. In the hygiene hypothesis, the Th1/2 paradigm of adaptive immune responses provides initial protection. However, any association between childhood infections and allergic disease has offered conflicting results. Therefore, the role of *H. pylori* infection in childhood in protecting against allergic asthma remains unclear. Persistence of an infection by *H. pylori* in childhood could cause more serious health problems later in the child’s life that offset any potentially protective effect against allergic asthma and its complications (5, 63–65).

*H. pylori-*induced inflammation causes changes in the gastric hormonal levels. The gastric hormones, leptin, and gastrin, have shown immunomodulatory activities. Gastric colonization of *H. pylori* leads to production of these hormones and activation of immunoregulatory mechanisms (20, 66, 67). The autonomic nervous system could also be influenced by *H. pylori* and it could also play a protective role against asthma (68, 69).

### *H. pylori*-mediated mechanisms against allergic asthma

Previous studies have shown that there is a significant association between *H. pylori* infection and asthma risk. Some researchers have suggested that *H. pylori* infection might be useful to trigger a protective response against allergic asthma (62, 66, 70). It has been proposed that early exposure to *H. pylori* is an important mechanism to prevent pediatric asthma development (63, 71). Based on the hygiene hypothesis, within an unhygienic environment *H. pylori* infection has been associated with a low prevalence of asthma.

However, there are some contradictory evidence that indicates *H. pylori* may indirectly induce allergic asthma through an exaggerated type 2 immune response (72). In addition, some studies have found no association between positive *H. pylori* infection and allergic asthma (5, 46, 62). Other studies have shown that seropositive *H. pylori* patients have a reduced level of IgE. The results of some studies have implied that *H. pylori* infection significantly protects children less than 10 years old from allergic asthma (8, 20, 73). Zevit et al. reported that there is an inverse correlation between allergic asthma and *H. pylori* infection in childhood (38). However, Tsang et al. and Jaber showed that there is no correlation between allergic asthma and *H. pylori* infection in children, and Fullerton et al. showed that there is no correlation between *H. pylori* and pulmonary function tests (62, 74).

*H. pylori* status could be used as a marker for other infections or socioeconomic conditions. In children, *H. pylori* infection is inhibited and limited by antibiotics administered for non-respiratory tract infections during the first year of life, which subsequently leads to an increased risk of childhood asthma (71, 75, 76). Th1 responses and related type 1 cytokines including IFN-γ, IL-12, and IL-18 are stimulated and dominated by infectious agents (5, 35, 66, 77). On the other hand, HP-NAF inhibits the allergic Th2 response via induction of the polarization of Th1 lymphocytes and protects people against allergic asthma (53, 59). It has been reported that *H. pylori* infection in neonates elicits tolerogenic IL-18-producing dendritic cells (DCs), which subsequently protects the host against allergic asthma (78, 79).

During *H. pylori* infection, T-regs (CD25-positive regulatory T cells) inhibit the Th-2-mediated allergic inflammation and suppress the allergic response. HP-NAP contributes to production of IL-12 by activating TLR2. IL-12 drives the differentiation of T cells toward the Th1 phenotype. Therefore, HP-NAP administration could potentially inhibit the Th2-mediated bronchial inflammation associated with allergic asthma. HP-NAP as a structural compound from *H. pylori* could be an important candidate for prevention and treatment of allergic asthma (35, 52, 53, 66, 75, 78).

The presence of *H. pylori* is inversely associated with gastro-esophageal reflux disease (GERD). *H. pylori* could alter the risk for asthma by modifying the immune responses or through its effect on GERD. Previous studies have shown that GERD can trigger asthma, and asthma is a risk factor for development of GERD (77).

From 50,000 years ago until now, humans and *H. pylori* have co-evolved. *H. pylori* is a primary cause of gastric ulceration, lymphoma, and adenocarcinoma. It is an important risk factor for human health and threatens their GI health (61, 63, 75). The present of *H. pylori* can be a risk factor for GI and GERD initiation, which can be an asthma trigger for the respiratory system, and the absence of *H. pylori* can be a risk factor for asthma initiation (opposing results affecting its possible application as a preventative).

## CONCLUSION

Complete elimination of *H. pylori* (HP-NAP) may lead to an increased prevalence and severity of allergic asthma. On the other hand, asthma may be associated with GERD and eradication of *H. pylori* might also reduce the risk of GERD and protect the host against asthma. According to the hygiene hypothesis, *H. pylori* has a potential protective role (as a bacterial infection) against asthma. High loads of *H. pylori* are not always indicative of a serious disease. There is not any accepted definition to distinguish between a pathogenic and non-pathogenic status of *H. pylor*i’s presence within a host body. New standards are required to be defined for various age groups and various conditions (some factors such as gender, age, and family history, etc.). *H. pylori* status could be one of the measurable risk factors for allergic asthma in children. The products of this bacterium can also be used for medical purposes. The genetic variations predisposing the host to *H. pylori* infection, strain-specific virulence factors, and pro-inflammatory markers in *H. pylori* infected patients with asthma need further evaluation. More focused studies should be conducted to clarify the exact mechanisms of action of *H. pylori* in the prevention, development, and treatment of allergic asthma.
